# Book Reviews

**Published:** 1828-09

**Authors:** 


### CRITICAL ANALYSES.

Quæ laudanda forent, et quæ culpanda, vicissim

Illa, prius, cretâ; mox hæc, carbone, *notamus.*—Persius.

*A Series of Observations on Strictures of the Urethra; with an Account of a new Method of Treatment, successfully adopted in Cases of the most obstinate and aggravated form of that Disease. Illustrated by Cases and a Plate.* By Richard Anthony Stafford, Member of the Royal College of Surgeons, and lately House Surgeon to St. Bartholomew's Hospital.—8vo. pp. 159. Longman and Co. London, 1828.

It is not the intention of Mr. Stafford to enter into minute details concerning the various kinds of stricture of the urethra. His chief object is to introduce to the notice of the profession a new method of treating the more obstinate and aggravated forms of this complaint; a department in surgery which, he considers, has hitherto been very defective.

It is to permanent stricture more particularly that the author directs our attention. That part of the subject, however, is introduced by a few observations upon the more simple and easily remediable forms of the disease. It is well known that Mr. Hunter divided strictures of the urethra into three different classes,—the permanent, the mixed case, and the true spasmodic. The latter form arises from the whole or part of the canal of the urethra being so highly irritable that the slightest stimulus will cause it to contract, and occasion the stream of urine to be suddenly obstructed. The disposition to spasm exists according to the constitutional excitability of the patient, or to the peculiar state of the canal, and it usually occurs in that portion of the urethra which, under natural circumstances, is the most irritable.

"Spasmodic strictures are very constantly the result of faulty digestion; and this is explained from the morbid irritability of the stomach extending its influence and sympathies to all other structures. Hence I have known a spasmodic stricture follow from eating of high-seasoned dishes and indigestible food, such as pastry, &c.; or from drinking subacid liquors, such as champaign, cider, &c.; and the proof that the spasm, in these cases, depends solely upon this cause, is shewn from the fact that, if the irritating substance be carried of, or the acid neutralised, the spasmodic state of the urethra ceases. In some persons the mere acrimony of the urine will bring on this affection. Faulty digestion, and many other causes, may produce this acrimony; and there are few of us who do not, at different times, experience changes in the secretion of this fluid. It at one time shall be limpid and unirritating, and at another so heated as to cause a burning sensation as it makes its exit. This irritating quality arises partly from the salts of the urine being in greater quantity, and partly from there being less fluid to hold them in solution. If these two causes are combined,—namely, the predisposition from increased general irritability of the system, and a concentrated state of the urine,—we cannot be surprised if the urethra be excited to inordinate contraction, and the patient suffer from spasmodic stricture. Besides this stimulating condition of the urine, where the general irritability exists, any other local cause will occasion stricture. Hence a bougie may become this cause; and we find at one time it will pass freely, while at another it will be arrested. The force of the contraction will also vary: in some cases, a steady and continued pressure for a short time will overcome the spasm; in others, the stricture will resist it altogether, although the force employed to pass the bougie shall have been sufficient to contort it." (P. 3.)

Much discussion has arisen as to the manner in which this contraction in the urethra is produced. It has been said by some writers that it never takes place in a situation anterior to that part of the urethra which is not surrounded by the accelerator urinæ, or ejaculator seminis, the sphincter vesicæ, the compressor prostatæ, and the levator ani muscles; and that these alone are the cause of the contraction. By others, spasmodic stricture is asserted to occur in the other parts of the canal, in muscular fibres which they have supposed encircle the urethra throughout its whole length. It appears to Mr. Stafford that the texture of the urethra is so very minute that it would be difficult to form any accurate opinion of its real structure, and consequently of the causes of spasm in this canal.

" It is sufficient to know that it possesses a contractile tendency to an extreme degree; and, as far as my own experience goes, I should conceive that the whole of the urethra may be affected by spasm, but that it is more active at that part of it where it is surrounded by the muscles necessary to the performance of its natural functions." (P. 7.)

In a permanent stricture, the contracted portion may appear, as Mr. Hunter expresses it, merely " as if the part had been surrounded by a piece of packthread."* It may be formed only on one side of the passage, or it may extend from one to two inches, and even farther, along the canal.

" The former of these, where the part appears as if it were surrounded by a piece of packthread, is the simplest form of permanent stricture. In this variety the membrane appears to be

* Hunter on Venereal Diseases, p. 113.

protruded forward into the canal by the parts immediately beneath it having become enlarged and indurated from deposition of lymph, without the membrane itself having participated in the change of structure. In this description of stricture, the contraction entirely encircles the urethra; but there is another variety, where it exists only on one side of the passage; and thus a duplicature of membrane, with a hardened base, juts forward into the canal. This sort of stricture exactly resembles the one that encircles the urethra, excepting that it occupies a portion, instead of the whole circumference of the passage; and it seems to correspond with what M. Ducamp and the French surgeons term a 'bride.'* Brides in the urethra, according to M. Ducamp, are usually supported by a large base, vascular, and jutting out in the canal, evidently formed by the mucous membrane, thickened from repeated inflammations. This description seems to answer to those contractions which take place on the side of the canal; and it appears to me that they are formed in the same manner as the circular stricture; but the parts around become enlarged, and the membrane is pushed, as it were, before it.

"The contractions, which occupy a considerable extent of the urethra, are generally extremely irregular; and their structure approaches to that of cartilage, being indurated and tough. In these cases, which are usually of long standing, the membrane likewise partakes of the change: it is firmer and thicker than natural; and should the diseased part be so situated that it can be pressed between the fingers, it feels as if a piece of whipcord or catgut were placed in the urethra, perfectly unyielding to the pressure. The passage, at the thickened portion, is so much obstructed, that it will scarcely admit through it the slenderest bougie. The urine can only be filtrated through it in so small a quantity, that it flows from the penis drop by drop, or in a stream not larger than a hair; and the canal at the part is so unequally thickened, that it is rendered tortuous." (P. 9.)

After having adverted to the various opinions that have been entertained as to the manner in which a permanent stricture is formed, Mr. Stafford states his own view of the subject.

"It appears to me that permanent contractions of the urethra are produced in the same manner as permanent contractions of other mucous canals, the œsophagus, the intestines, &c. (with the exception of scirrhous contractions;) that a continued chronic inflammation having existed for a considerable time in the part, its vessels are enlarged, and lymph is very gradually deposited in its interstitial structure. This slowly becomes organised and con-

* "Elles sont parfois supportées par une base large, vasculeuse, saillante dans l'interieur du canal, évidemment formée par la membrane muqueuse, épaissée par des inflammations répétées."—Ducamp, *Traité des Retentions d'Urine*, p. 13.

densed; and by its increase the canal, in progress of time, is entirely blocked up. The membrane, therefore, and the part immediately surrounding it, are rendered more and more indurated, in proportion to the length of its continuance, till it even assumes a hardness belonging rather to cartilage than any other structure." (P. 15.)

Numerous causes may give rise to the formation of permanent stricture. Whatever will produce inflammation of any particular portion of the urethra, whether it has a specific or a spontaneous origin, will equally tend to cause an alteration of structure at the affected part. Gonorrhœa is the most common foundation of the disease. After the first stage of it, a chronic form of inflammation remains, which is peculiarly favorable to that deposition of lymph and thickening of the parts which is the cause of the canal being afterwards permanently narrowed.

"This seems to be exemplified by the circumstances attending gleet, for it frequently happens that when this discharge has continued for a considerable time, it is kept up in consequence of the formation of a stricture. During the time I was house surgeon at St. Bartholomew's hospital, I had frequent opportunities of examining patients who were suffering from a gleet of one or two years' standing, and all the remedies used had been ineffectual. When a bougie was passed, a stricture was usually discovered in some part of the canal.—Another cause of the formation of permanent stricture is the employment of too-powerful astringent injections in the cure of gonorrhœa. These, no doubt, will stop the discharge; but they induce at the same time a chronic inflammation in some part of the urethra, which at length terminates in a permanent contraction." (P. 17.)

Upon this point there is some difference of opinion. Mr. Hunter, for instance, declares that he has found stricture exist as frequently after gonorrhœa where no injection had been used, as where they had been employed.

After a brief examination of the causes of permanent stricture, and those forms of disease to which the urethra is most liable, Mr. Stafford proceeds to describe the most usual situations of permanent strictures, their symptoms, and their consequences.

Chap. 2d.—Strictures have been known to form in every part of the urethra, excepting in that portion of it which is surrounded by the prostate gland. Their most usual situation, however, appears to be where the canal is narrowest.

"Thus they are most frequently met with at the entrance into the membranous portion, immediately behind the bulb, in the membranous portion itself, and about four inches and a half from the orifice. These, according to measurement, are the most confined parts of the urethra, and it is probable that they are more liable to become strictured on account of their being exposed to the stream of urine, from their protrusion into the canal. In the same manner we find strictures of the œsophagus to occur where the funnel of the pharynx narrows into the gullet; strictures of the cardia, where the passage is straitened by the muscular fibres of the diaphragm; again, at the pylorus, where the bag of the stomach contracts into the duodenum; and, lastly, in the rectum, where the sigmoid flexure turns over the ridge of the sacrum. At these different points the canal receives the impulse of the contents as they pass, and, if predisposed, are excited to inflammation, ending in thickening and stricture. In a similar manner, in the urethra, the more contracted parts receive the momentum of the stream of urine, and, if predisposed, become inflamed, and, when once inflamed, the same causes continue to keep up that state, and to aggravate the affected part. This I am inclined to think is the cause why strictures more frequently occur at particular points. But there are other situations, also, where it is not uncommon for strictures to exist: the orifice itself is often contracted, and the part three inches and a half distant from it. Mr. Hunter considered that the bulb itself was most liable to the attack: I am inclined, however, to agree with Mr. Macilwain, that the affection most commonly occurs immediately behind it; and this I am led to infer, both from experience and also from comparing together the specimens preserved in our museums." (P. 25.)

Those who are afflicted with strictures are occasionally attacked by shooting pains in the perineum; they are subject to nocturnal emissions, a gleety discharge, attacks resembling gonorrhœa, and a constant desire to make water. "Sometimes they have a fluttering sensation at the strictured part; sometimes a cluster of vesicles, which have been called 'herpes præputialis,' followed by ulcers, will make their appearance upon the glans penis, without any apparent cause, just as vesicles and ulcerations break out about the mouth, indicating an irritable and inflamed state of the mucous membrane of the alimentary canal; and sometimes great irritation may subsist at the orifice of the urethra." (P. 30.)

More serious symptoms occasionally depend upon the presence of stricture. The semen is prevented from making its exit at the time of coition. When this is the case, it is thrown backwards towards the bladder, and makes its exit some time afterwards. Swelled testicle, it is well known, is often a concomitant symptom. When a patient has long been afflicted with strictures, the urine frequently passes away involuntarily, and he has a constant desire to make water. Numerous morbid changes occur in the urinary organs from obstruction in the urethra. Mr. Stafford has known a fistulous passage to extend from the urinary canal to the back part of the thigh.

"By these fistulous passages, the urine, at the time of micturition, makes its escape, instead of through its natural channel; and, when they have continued for a long time, their sides become hardened; and in some instances they are lined by a kind of membrane. Specimens of this description are to be seen in the College of Surgeons. In one case there are two fistulous passages, which resemble regular mucous canals, being lined by a membrane analogous to the mucous tissue. It is a curious fact that, if a false passage be made, leading from one part of the urethra to another, and the urine passes through this new channel, it is also found to be lined by a membrane, or what looks like a membrane, and it has the appearance of a natural-formed canal. This I have seen in one or two instances; and my friend Mr. Lawrence mentioned to me a case where he found, in the urethra of a gentleman who had been in the habit of having bougies passed, a new canal formed, of between two and three inches in length, commencing anterior to the bulb, running close along the side of the natural canal, and terminating in the prostatic portion. This canal had a smooth mucous surface, very similar to the urethra itself. In a case also which occurred to myself, the urethra, in that portion of it which passes through the penis, was impervious; but immediately under it there was a newly-formed passage, which likewise was lined by a membrane of the same description." (P. 39.)

*Treatment of Spasmodic and Inflammatory Stricture.*—Mr. Stafford observes, that "stricture of the urethra has been, and still is, considered by many surgeons as a mere mechanical obstruction, without the least reference to it as a disease produced by inflammation. If a bougie can be passed through the stricture, it is sufficient: no further treatment is considered necessary." We really believe that there are very few surgeons who are amenable to this accusation; and still fewer who require to be informed that "other means can be employed,—such as local bloodletting, soothing the parts, and attention to the general health." The subsequent observations upon the subject of treatment are very judicious, but, as the substance of them may be found in most works upon the same subject, it is not necessary we should dwell upon them.

The next chapter is on the treatment of permanent stricture, which we have seen is the principal object of the work. In cases of permanent stricture, we have to contend with a part of the urethra irregularly thickened, and so indurated as to resemble the structure of cartilage; and with a narrow canal, contracted to that degree, and so extensively, that it is either quite impermeable, or it will only admit through it the smallest-size bougie.

"To effect a cure of these states of the urethra, we have to enlarge the contracted passage; to procure the absorption or destruction of the surrounding thickened tissue; and to restore the parts to their healthy condition.

"The different plans which have been adopted to permeate this description of stricture, and to restore the urethra to its healthy condition, are four in number. First, it has been attempted to make the part ulcerate by the continued pressure of a bougie upon it. Secondly, some surgeons have endeavoured to force through the contraction with a conical sound. Thirdly, caustics have been applied to the diseased part, with the view of destroying it. And, fourthly, the part has been divided from the perineum. The two former of these plans of treatment, the endeavouring to make the part ulcerate, and the forcing through the contraction with a conical sound, are now, from the danger and uncertainty with which they are attended, totally relinquished; and the two latter, the applying caustic, and the division of the stricture from the perineum, are the only means which are at present practised. These, however, are also attended with great risk. The destruction of a permanent stricture by the application of caustic, is an extremely tedious and painful process, uncertain as to its final result; and at the same time, as will presently be shewn, symptoms frequently arise that endanger the life of the patient. The division of the stricture from the perineum is a very difficult and painful operation: it is often unsuccessful; and, also, it is so little susceptible of being reduced to fixed rules, that it can hardly become a measure of general adoption." (P. 63.)

With respect to the treatment of stricture by caustic, its most strenuous supporters admit that it is often followed by alarming symptoms. Sir EVERARD HOME mentions many of these ill effects, and, amongst others, that very profuse hemorrhage is sometimes caused by the application of lunar caustic. He relates six cases, in all of which the patients were much reduced by loss of blood, and some of them bled so profusely that the quantity lost amounted to two or three pints. When this mode of treatment does succeed, it is frequently very tedious. The potassa fusa is said to be still more objectionable.

Having pointed out the imperfections of the more common methods of treating this form of stricture, Mr. Stafford proceeds to explain the plan which he has adopted.

"It is the division of the diseased part within the canal of the urethra. The advantages of this mode of treatment are, that it effects with certainty, and in a short time, what the caustic is intended to accomplish by repeated and tedious applications; and it is free from the difficulties of the operation for the incision of the stricture (an operation little less painful than that for lithotomy,) through the perineum, thereby saving the patient the inconvenience and misery of a new channel, leaving but little for nature to repair; and at the same time allowing the urine to flow through its natural passage. For this purpose I have invented two instruments, the one to divide permanent strictures, while yet a small bougie or wire can be passed through them; and the other to divide those strictures which are impermeable.

" The instrument for operating on permeable strictures (which, for the sake of distinction, I have called the Double Lancetted Stilette,) consists of a round silver graduated sheath, open at both ends, of the size of No. 10 catheter, with rather a less curve, and of a stilette, which is also hollow, and open at both ends. This stilette is furnished, at one end of it, with two oblong lancets; and at the other with a handle, resembling a button. When the instrument is complete, the stilette fits into the sheath, so that by pushing the handle the lancets will project from the extremity of the tube, and by drawing it back they will retire into it again. When used (the mode of doing which will be presently explained), the instrument is passed over a wire down to the stricture, and the lancets are thrust forward on each side of it, by which the contraction is made as large as the natural size of the urethra.* The armed stilette, intended to divide impermeable strictures, exactly resembles the one just described, excepting that, instead of the stilette being hollow, it is solid, and in the place of two there is only one lancet.

" Before using the instruments, the exact distance of the stricture from the extremity of the urethra should be ascertained. In the armed catheter, which is intended to divide strictures over the wire which serves as a guide, the wire must be introduced through the stricture first. The mode of accomplishing this is by passing the smallest possible sized catheter, made to contain the wire, into the bladder. The wire, which is double the length of the catheter, and blunted at one end, so that it may not injure the bladder, is then pushed forward, and the catheter gradually withdrawn, by which the former is left in the canal of the urethra. The armed catheter is then passed over the wire, until its point rests against the stricture, (which is known by means of the graduation,) and, being held securely in such position, the handle of the stilette is pressed gently and gradually. As soon as any impression is made, the lancets should be allowed to retire into their sheaths, and the blunt point of the instrument urged forward. If it do not pass on, the lancets may be again used as before. After the stricture is divided, the armed catheter should be withdrawn, and its place

* " This handle has hitherto been formed like a button; but I have thought it would be of advantage to have it made like two rings, large enough to admit the finger and thumb, similar to the handle of a pair of scissors."

supplied by one of elastic gum, of the same size. This should remain for a day or two, to prevent the reunion of the divided parts, and to preclude the possibility of extravasation of urine; and, on its removal, a bougie should be passed twice in the week, or as often as may be judged necessary, for some time; and the same treatment adopted as for stricture in general. The armed stilette, intended to divide impermeable strictures, must be used precisely in the same manner as the other, of course excepting the wire, which cannot be introduced; and the same directions for the after treatment are necessary for both.

"In some of the cases in which the instrument has been employed, the division of the stricture has been followed by more or less inflammation, but seldom amounting to a great extent. Such an occurrence should be guarded against by the application of leeches to the perineum immediately after the operation, and by a strict adherence to the antiphlogistic regimen. If the presence of the catheter that is left in the urethra cause considerable pain, it must be withdrawn; but in this case it is of material consequence to pass a bougie daily, lest the divided parts reunite.

"It may be objected to the use of these instruments, that there is a liability of making by them a false passage. This is prevented, in the permeable stricture, by the wire acting as a director, and limiting the incisions to the size of the natural canal, so that it is impossible to deviate from the course of the urethra. With regard to the second case, or when the stricture is not permeable, it must be admitted that, in unskilful hands, or by violent means, a false passage may be formed; but that, with common care, this is not likely to occur, is proved by the result of not less than twelve cases of impermeable stricture, where I myself have used the armed stilette, or have seen it employed. In no case was there any false passage made. I very much question, however, if the same number of cases had been treated by caustic, whether they would have been attended by the same success. On the whole, therefore, it may be safely inferred that, although it is possible that a false passage may be made by the single lancetted stilette, yet with common care it may be avoided. It might, perhaps, also be thought that considerable hemorrhage would follow the division of the stricture by these instruments, and that they would produce much pain; but, in the cases in which it has been employed, the bleeding has been inconsiderable, and the pain trifling." (P. 70.)

The author has never had an opportunity of examining the parts after the use of his instruments, but he presumes that the urethra is afterwards restored to its healthy condition; for, in passing the catheter or bougie at different periods after the operation, he never found any hardness at the diseased part. It is not only in the urethra that Mr. Stafford has remarked that the division of an indurated structure will cause its absorption:

"In one instance, where Mr. Lawrence operated for the stone, there was an extremely enlarged and hardened prostate gland (so hard that it resembled the structure of gristle); and this, some time after the operation, became of its natural size and structure. In a case, also, where Mr. Titus Berry divided a hardened contraction of the œsophagus with the armed stilette, the part was restored to its natural character. It is almost impossible to say, for certain, by what process this is effected: it is, nevertheless, a very valuable fact, and one which is well worthy the notice of the profession." (P. 79.)

The cases in which the armed stilette appears likely to be most beneficial, are those where the contraction is so hardened, and of so unyielding a nature, that it will not admit of being dilated at all, or where we can only obtain a temporary cure.

"In these cases the division of the diseased part would be of great advantage; for, by one incision of the armed stilette, the contracted portion would be made as large as the rest of the canal; and, at the same time, (at least judging from the cases where strictures of this description have been already divided by this instrument,) the induration would completely disappear in the course of a month or six weeks, or even less time, and the stricture would be permanently cured." (P. 81.)

In cases of partial and total retention of urine, the armed stilette would be found particularly serviceable, and in all those instances where there has been extreme difficulty in passing a bougie or catheter, the double lancetted stilette would at once relieve them. "In retention of urine, also, where the stricture is quite impermeable, it certainly must be allowed to be far better to divide it with the single lancetted stilette, than to puncture the bladder."

Fifteen cases are detailed in which the instruments were employed. In one only they were unsuccessful. In this instance the patient died; but we agree with the author that it appears more than probable that the fatal termination of the case could not be attributed to their use. From the experience which Mr. Stafford has now had of the lancetted stilettes, and from the success which has attended their use, he hopes that they may, in some cases, supersede the necessity of more serious operations; and, in others, afford relief with greater certainty than the means at present employed.

It is not for us to speak dogmatically upon a subject with which we are not practically acquainted. We cannot, however, feel quite confident of the entire safety of these instruments, although we admit that there are cases in which we might be inclined to use them, upon the principle of *de minima malis.*

Mr. Stafford once in the course of his work slightly mentions his belief that the French and German surgeons have attempted the division of strictures within the canal of the urethra, "but by what means, or with what success," he is unacquainted. The practice is certainly not new; but we are indebted to Mr. Stafford for having improved upon the instruments* which have been employed for the same purpose by Amusat and others.

---

*Cases of Mental Disease, with Practical Observations on the Medical Treatment. For the use of Students.* By Alexander Morison, m.d. President of the Royal College of Physicians, Edinburgh; Member of the Royal College of Physicians, London; Lecturer on Mental Diseases, &c.—8vo. pp. 164; with Plates. Longman and Co. London, 1828.

The object of this elementary publication is to present to students a collection of cases of ordinary occurrence, in which the medical treatment usually employed is detailed. By the advocates of craniological phrenology, an attempt has been made to shew that the different kinds of partial insanity are dependent upon different morbid states of particular convolutions of the brain, in which Dr. Gall says the different propensities and affections reside, and they direct topical treatment to the supposed diseased organ. But, if we examine the brains of those who have laboured under monomania, we find the proofs of disease, such as inflammation or its consequences, existing in more than one convolution, and diffused over the membranes covering them. The arrangement proposed by Pinel and Esquirol, founded on the morbid manifestations of the mental functions, appears to Dr. Morison better suited to the present state of our knowledge. In proof of the applicability of this mode of arrangement, he states that, in a collection of nearly three hundred cases, taken indiscriminately with a view to ascertain this point in regard to practical purposes, he has found little difficulty in assigning to each a definite place in it.

The author first makes a few observations on the general principles upon which the medical and mental treatment are founded, before he proceeds to the detail of cases. "In every case of mental derangement, it is presumed that more or less corporeal disorder exists. Hence the propriety of dividing the treatment into medical and mental, or, as it has been usually termed, moral."

* The instruments are made by Fergusson, Giltspur street, St. Bartholomew's Hospital.

Our readers are probably aware that a few writers contend that mental derangement may exist without any corporeal affection. We have, however, no doubt that the opinion expressed by Dr. Morison is perfectly correct. We have so frequently touched upon this subject, that it is unnecessary we should again cite any particular authorities in corroboration of the doctrine maintained in the present work. They who have had the most experience in mental diseases, and whose works betray the least disposition to indulge speculation in opposition to facts, agree that mental disease presupposes either disease of structure or derangement of function in some part of the body.

"In the employment of the former, we are directed by indications, presenting themselves, to counteract the various deviations from the healthy state which may occur in the corporeal functions. The first object of inquiry is the origin of disease. In every case where the mind is disordered, it is now generally admitted that its organ, the brain, is either primarily or secondarily affected; probably not so universally the former as some late authors contend. Still, in every case, our attention must be first directed to investigate its probable condition, which varies in different cases. In some, the irritation of this organ attending the mental derangement is inflammatory; in others, a state of active congestion, or fulness of blood-vessels, without inflammation, prevails. This fulness, again, may be of a passive description, depending upon a semi-paralytic dilatation of the cerebral vessels. With a view to obviate these morbid states of the blood-vessels in the head, the abstraction of blood generally or locally,—the application of blisters,—the insertion of issues,—and the application of cold, are all indicated, more or less, in different cases, and upon rational principles, as well as other evacuations tending to diminish determination of blood to the head; and, to remove the effects of these morbid states of the vascular system, such as thickening of the membranes, depositions of serum, &c., certain remedies, supposed to excite absorption,—among others, mercury, diuretics, and local stimulants and drains,—have been employed.

"The influence exerted upon the brain by disorders existing in other organs, leading to derangement of its functions, appears to be intimately connected with the state of the nerves and ganglions of the great sympathetic nerves, supplying the organs of digestion and of generation. The unusual sensations experienced in the abdomen leading to erroneous ideas respecting their nature, so common in some varieties of insanity, as well as those occurring in epilepsy and hysteria, are, it is probable, phenomena of a deranged state of this system. How great an effect slight irritation thereof may produce, is proved by delirium and convulsions, symptoms dependent on the nervous system, including the brain, being produced by worms in the intestines irritating the extremities of these nerves, without any reason to suppose inflammatory action.

" Where abdominal irritation, then, may be supposed to exist, the employment of remedies acting upon the stomach and bowels is rationally indicated, and they are of extensive utility in mental disorders. The connexion of the genital organs with mental disorder is likewise well ascertained. In females, menstrual irregularities and other uterine affections; in males, onanism and excessive venery, are frequently followed by or attend upon insanity. Hence the good effects sometimes produced by the re-establishment of the menstrual, the occurrence of the hemorrhoidal discharge, and the removal of debility; and the propriety of employing medical treatment corresponding to these indications.

" With regard to the nervous system itself, it does not appear irrational to suppose that regular distribution or congestion of that agent, which is the material vehicle of sensation, may take place in the nerves, that this ascendant fluid may flow too rapidly or accumulate too much in certain parts of the nervous system, independent of sanguineous disorder, and produce increase of general sensibility and of muscular irritability, giving rise to painful and unusual sensations, the cause of those sudden delusions, and of those violent and irregular movements, so common in the insane. To mitigate or subdue those, recourse is had, and upon rational principles, to the soothing properties of the warm bath, and of narcotics of different kinds, and to the invigorating effects of tonics.

" It is in directing the mental or moral treatment, however, that the arrangement, founded on the diversity of the mental phenomena, is chiefly useful; for cases arranged under the same head, and requiring similar mental management, may require very opposite medical treatment.

" In order to conduct the mental treatment with efficacy, the most important object is to obtain full information of the patient's previous history, and particularly of the mental cause giving rise to, or at least intimately connected with, the production of the disorder. Such may be excessive application to business or to study, political anxieties, commercial difficulties, religious doubts, disappointed affections, remorse of conscience, and various passions. Possessed of such knowledge, we are the better able to appreciate the phenomena of his delirium, the association of his ideas in general, and the tendency of those ideas on which his mind chiefly dwells, thereby foreseeing and preventing mental irritation, removing or diminishing uneasy sensations, and lessening the frequency of fits of fury or of despondency.

" On remissions occurring, we are the better able to direct the patient's attention to subjects least likely to agitate him, avoiding those on which the train of erroneous ideas or delusions depend. By ascertaining and applying what is most wished for, or making him avoid what is most dreaded, we are the more enabled to exercise with judgment the opposite emotions and affections that may be suitable to the different kinds of partial insanity.

"In such cases, then, where mental treatment is applicable,—for in furious madness seclusion, medical treatment, and adequate restraint, and in chronic dementia and idiotism safe custody and kind treatment, only are required,—the leading indication is to diminish and remove delusions or erroneous ideas, by exciting the attention, and by withdrawing it from favorite but hurtful subjects of thought. With this view, recourse must be had to occupation exercising the body or employing the mind, or both, by such means as labour of various kinds, active or sedentary amusements, walking, riding, travelling, music, drawing, reading, &c. In general, it may be observed that a daily round of easy occupation, not attended with danger, ought to be established, in which the patient may be employed, as, in the discretion of the physician, may seem best adapted to his former habits and his present state. Where numbers of patients are assembled, the treatment to be observed necessarily requires a judicious classification; for it would be highly prejudicial to allow those who have their particular dislikes, those who may awaken distressing ideas in others, or those who may strengthen each other's delusions, to be together.

"The excitement of certain emotions or passions is sometimes of use in mental treatment: in particular, the agreeable emotions of hope and of religious consolation, and the disagreeable ones of shame and of fear. To excite the latter in a moderate degree, certain mechanical means have been employed, as the rotatory machine and the douche of cold water; and they have been sometimes employed with advantage. A few cases are on record where dexterously humoring the patient's delusion has been successfully tried; but these are so rare that little dependence is to be placed in that mode of treatment. To conclude, it must be kept in mind that no general rules of mental treatment can be laid down applicable to every case. Each patient must be studied individually, in order to acquire such knowledge of his mind as to enable us to control and regulate its operations." (P. 4.)

After these brief general remarks, the volume consists entirely of a detail of fifty-one cases of various species of insanity, upon each of which the author offers short observations. They are all interesting as practical sketches, although we perceive no particular novelty either in the general course of the various shades of mental disease which are described, or in the treatment which was adopted. We shall select a few of the most interesting examples.

"*Intermittent Mania.*—A. O. H., married female, æt. thirty-three.

"June 12.—About a month ago she shewed symptoms of mental disorder, being five months after childbirth. The first sign of it was her attempting to go out by the window, having previously taken leave of her children. Afterwards she expressed aversion to them, to her house, and to every thing about it, with a continual desire and attempts to wander about.

"At present her disorder assumes a periodical form. In the morning, as soon as she awakes, she begins to talk incoherently, and is incapable of restraining herself: she continues in this state until noon, or a little longer. In the evening she is perfectly calm and collected. This is the first attack of insanity under which she has laboured, and there is no reason to suppose that she has a hereditary tendency to it. The failure of her husband's circumstances preceded its appearance.

℞. Pulv. Rhei gr. xv.; Calomel gr. ij. M. sumat statim, et pro re nata.

℞. Pulv. Cinchonæ ℨj. sextis horis abs. parox.

"25th.—Has made rapid progress towards recovery.

"July 5th.—Is desirous of returning to her home.

"23d.—No signs of mental disorder remain.

"In this case the usual treatment of intermittents was eminently successful." (P. 51.)

Cases of *Demonomania* are by no means common. Women are more frequently affected with it than men. Two or three cases have fallen under the observation of the author. Esquirol has met with several; in one of which a woman conceived that the devil had run away with her former body, of which her present one was only a shadow. She endured the most dreadful uneasiness,—was in fear of eternal damnation night and day,—conceived herself surrounded by flames of brimstone,—and heard devils within her disputing who should possess her. These horrible ideas, as might be expected, deprived her of sleep and appetite. She was likewise in the habit of beating herself severely. The sensibility of her skin, however, was so much blunted, that pins could be thrust through it without her seeming to feel them.

*Case of Demonomania.*—"A. G. O., married female, æt. fifty.

"Feb. 2d.—About five months ago she became insane, for the first time. Her disorder at that time manifested itself by extreme melancholy, and by talking to herself incoherently. In this state of depression she continued till about a fortnight ago, when her disorder assumed a more active character: if contradicted or opposed, she was much agitated, and occasionally violent; her actions were irrational, and she frequently talked of royalty; but her present conviction is that she had sold herself, her husband, and her son, to the devil, and that she is therefore excluded from divine mercy.

"She has not attempted to commit violence upon herself or upon others, and there is no reason to believe her complaint to be hereditary.

"She has occupied herself much in reading old religious books.

℞. Pulv. Rhei gr. xv.; Calomel gr. ij. M. statim et p. r. n.

"12th.—℞. Antimonii Tartar. gr. ij. omni mane.—℞. Camphoræ gr. v. Extr. Hyoscyami gr. v. M. forma pil. h. s.

"22d.—No change.

Omitt^r Antim. Tartar.—℞. Pulv. Serpentariæ ℈j. ter die.

"March 1st.—Is now apprehensive respecting her own health, and has absurd notions about the state of her abdominal viscera." (P. 75.)

The following case is worthy of notice, on account of the observations Dr. M. makes on the use of camphor.

"A. J. G., female.

"August 21st.—Has been several years in a state of insanity, the prominent feature of which is unfounded fear and alarm, with melancholy. She likewise says that she has lost all the feelings of a human being, and resembles a brute, and that she cannot feel towards her children as she should do. She is weak in body, and has rheumatic pains in her back.

℞. Sp. Camph. ℥j.; Liq. Ammon. Carbon. ʒiij. M. fiat P. linimentum dorso applicandum.—℞. Camphoræ gr. x. bis quotidie.

"Oct. 9th.—The use of camphor has materially added to her comfort. She says that she has much less anxiety than she used to have, and expresses her gratitude.

℞. Camphoræ gr. x. quater in die.

"Dec. 14th.—Is materially improved; feels herself comfortable, and is more able to work than she has been for years.

℞. Camphoræ gr. x. sexties in die.—℞. Infus. Cascarillæ ℥jss.; Acid. Sulph. dilut. m. xx. M. bis quotidie.

"It is conceived that decided advantage has been derived from the use of camphor, which was continued to the extent of nearly ℈iv. daily: the serenity of her mind, after so much perturbation, was remarkable. Under the use of it she continued to improve, became very orderly and comfortable. After some months the dose was gradually decreased, without change of symptoms, and it was left off entirely by substituting bread-pills, that her mind might be satisfied during the weaning her from it. In July, medicines were discontinued, and a year afterwards she continued well.

"The mode of operation of camphor is not yet well understood: it generally increases the heat of the skin, and in large doses appears to be powerfully sedative. The late Dr. Alexander, when making experiments, nearly killed himself by taking ℈ij. at once, which he fortunately was made to reject by vomiting produced by warm water. It has been much used in mental diseases: Aenbrugger recommends it particularly when the pulse is slow, the countenance pale, the hand cold, contracted, and trembling; and, in men, when the genital organs are cold, the penis retracted, and the testicles drawn up towards the pubis!

" Dr. Perfect made use of it in large doses, such as ℈ij. for a dose; but it must be confessed that, though we now and then find a cure apparently produced by camphor, it is but seldom. In the above case, it is true, camphor was exhibited successfully, and in very large doses; and still larger are upon record. Dopson says he gave a furious maniac ʒiij. of camphor in the course of twenty-four hours, ℈j. at a time; and during the following day the same quantity; and that a perfect cure was accomplished. Hufeland is said even to have injected camphor into the veins of an insane female, and to have cured her.

" We must not, however, forget the violent effects occasionally produced by large doses, as in the experiment of Dr. Alexander; and, indeed, that death itself has actually been produced by them." (P. 85.)

In cases of *Erotomania,* or partial insanity with love, the doctrines of Gall and others have led to the topical application of remedies to those parts of the head corresponding with the supposed cerebral organ within in a state of disease. Bleeding, in particular, is said to be very useful in this variety of partial insanity. The author admits that detraction of blood from the back part of the head is of service in cases where amatory excitement prevails: but he believes, and we think truly, that we shall find this to be the case in all the varieties of mental disease in which bloodletting is beneficial.

The use of opium in insanity is not so well understood as we could wish. It is by no means easy to discriminate the cases in which it ought to be employed.

" Vascular excitement is what chiefly deters us from its use; for, if there be inflammatory action or congestion of blood in the brain, opium may be productive of serious mischief by increasing these states, thereby exciting increased violence and fury.

" We must likewise attend to its effects of costiveness and diminution of the secretions, on which account the hyosciamus is in many cases to be preferred.

" All these considerations, however, are not to deter us from the proper use of this valuable remedy.

" When want of sleep occurs, which it so frequently does in insanity, after the necessary sanguine and alvine evacuations have been made, opium may be tried in most cases, especially if the disease has lasted some time.

" When the disease begins to subside, and the patient, beginning to convalesce, is kept awake by fear, jealousy, or suspicion, opium (if the above circumstances do not forbid) will be found of much service.

" Where grief and disposition to shed tears prevail, opium will frequently be of use; as well as in those cases connected with intemperance, particularly in delirium tremens, when the pulse is small and frequent.

"The quantity that may be given is greater than what those in a state of mental health in general can bear, but it is not safe to give a large dose at first: it is better to begin by a grain or two, and gradually to increase it. The largest quantity I have heard of being given, was in a case treated by Dr. Galloni, of Rheggio. This was a male patient, whose complaint, in the commencement, was treated as phrenitis by copious evacuations of blood, and who remained in a state of furious mania upwards of three years, during which time various sedatives, among others digitalis and hyosciamus, were given. At last a trial was made of opium. He began by giving one grain four times in the twenty-four hours, which he gradually increased to ten grains four times a day. Some abatement of the fury was produced, but, symptoms of dropsy appearing, the opium was discontinued. Some time after, the opium was again resorted to, beginning with the same dose of four grains in twenty-four hours, and gradually increased to 170 grains in the day! The result was, that his fury abated, his ideas became more coherent, he was induced to occupy himself in drawing, and a complete cure followed. The opium was left off in the same gradual manner." (P. 115.)

In many cases of cerebral disturbance, which may not amount to positive insanity, and in insanity also, we may certainly be justified in cautiously trying the effects of opium. In these instances, and where the mind is really deranged, we should prefer the Liquor Sedativus of Battley. Its sedative powers are as much to be depended upon as the tincture of opium, and it is infinitely preferable whenever we have reason to be apprehensive of increasing the actions of the vascular system. It also possesses the negative merit of rarely constipating the bowels. We have frequently found patients derive much benefit from the use of this preparation, who could never bear the tincture of opium even in the smallest doses.

In visiting private asylums for the insane, Dr. Morison has frequently observed that the difficulty of procuring proper machines for administering the douche, and rotatory motion, has prevented the medical attendant from making a trial of them. He has therefore given drawings of machines for each of these purposes, which may be erected at little expense by any intelligent carpenter.

Many good practical remarks are appended to several of the cases, the detail of which we have not thought it necessary to give. The author cautions us against too indriscriminate an employment of purgatives. The milder ones, and in moderate doses, in general succeed better than the very drastic purgatives. It is chiefly in the early stages of mania and monomania, where the strength of the constitution is undiminished, that calomel and jalap are most beneficial. The cold bath is unsafe, unless reaction takes place after it: hence the propriety of not continuing it too long. Considerable caution is necessary in the employment of emetics. When inflammatory action or congestion in the head prevails, these should be diminished before having recourse to emetics. Some insane patients bear very large doses of the tartar emetic. We remember a lady, whom we attended with Dr. Burrowes, who took ten grains of the tartar emetic three or four times, at short intervals, without any sickness being produced. It is prudent to begin with the ordinary dose. Frictions with tartrate-of-antimony ointment has been much employed in cases of mental disorder, especially by Dr. Muller of Wurzburg. The experience of other physicians does not confirm his favorable reports. Where the suppression of a cutaneous eruption has accompanied the mental disease, the frictions have appeared useful.

The variety of appearances observed in the encephalon of the insane is so great, that we are not yet able with certainty to draw many useful inferences from them. On the continent, attention has lately been directed to the state of the great sympathetic nerve and its ganglions; and morbid alterations, such as inflammation and induration of the latter, and increase of size, with induration, of the former, have been found, in cases of mania and imbecility, by Pinel, Tiedemann, and Autenrieth.

As a preliminary study, this little volume will be consulted with advantage. We cannot with propriety complain that the various subjects are treated in a superficial manner, as the author does not profess to give more than a practical sketch of the different species of insanity.

---

*A Rational Exposition of the Physical Signs of the Diseases of the Lungs and Pleura: illustrating their Pathology, and facilitating their Diagnosis.* By Charles J. B. Williams, M.D.—8vo. pp. 192. Underwoods, London, 1828.

This work is divided into two parts: the first contains an exposition of the general physical signs of a healthy and diseased state and action of the thoracic viscera, to which is prefixed a chapter on the properties, &c. of sound; the second comprehends the pathological history, and physical signs of the principal diseases of the lungs and pleura. At the end of the volume, tabular views are given of the physical signs, illustrated by a plate shewing the situation of the regions of the chest. A diagram of the stethoscope, and an accompanying explanation of the best principle of its construction, are also added. The author states very candidly in his Preface, that most of the facts which he has described have appeared in the works of LAENNEC and ANDRAL. Wherever his experience has not enabled him to give the same as the result of his own observation, he has referred to their competent authority. Where, in point of fact or opinion, he has differed from them or from others, he would modestly wish his dissent to be viewed rather as a question to be answered by others, than as in itself superseding former observations or opinions.

Chap. 1. *On the Physical Signs of Disease.*—By *physical signs,* Dr. Williams means such as depend on the direct operation of known laws of natural philosophy on our organs of sensation.

"As they are produced by the physical state or condition of a part, they become indications of that state or condition, as certain as the laws, of which they are exemplifications, are unerring and sure: and the physical state of a part of the body may be ascertained with more or less certainty, as its physical signs, or relations to these natural laws, are more or less appreciable by our senses." (P. 1.)

The number of diseases that come under the cognizance of vision is very limited, as by far the greater part of the body is excluded from its sphere.

"Derangements of the surface, and of the openings of some of the passages to the interior, can alone be subjected to the direct examination of the eye. Mediately, physical changes of internal organs can be perceived by signs only, when their size, form, or position is so far altered as to cause displacement of some external part; and the knowledge that such a sign gives us, although scanty, is often valuable." (P. 2.)

In the same cases, the sense of *touch,* or *tact,* will furnish us with further knowledge as to the form, substance, and constitution of a diseased part; and, when perfected by experience, may frequently discover organic changes that are altogether imperceptible to sight. In some cases, the sense of *smell* may assist us in diagnosis. In the last January Number of our Journal, p. 62, we gave, from HECKER's "Litterarische Ann. der gesammten Heilkunde," an interesting paper on this subject. The author of this communication, Dr. VOGEL, remarks "that if the changes in the odour of the breath were more carefully observed, the diagnosis of the physician might be improved, particularly in febrile diseases."*

* It is said that a practitioner in Berlin, from having a very acute smell, is frequently enabled to determine the particular species of exanthematous dis-

"Sound, as it may be both generated and propagated in every form of matter, solid, liquid, and aeriform, may be therefore considered a mean of examination of parts removed from sight and tact, more promising as its sphere is less limited. It is requisite, however, that the object of examination be capable of producing or transmitting audible sound; and that changes in the part produce corresponding changes in sound thus produced or transmitted, that may be appreciated by the ear. The relations of the organ of hearing to the qualities of external objects are, in ordinary life, much less exercised than those of tact and vision. Yet continual experience proves to us that the substance or consistence of simple objects is, in some measure, declared by the sound which they emit when struck. The sound of liquids in contact with air is familiarly distinguished from that of solids in the same medium, and a little more attention discovers the varied sounds which air in motion produces in contact with solids of different forms." (P. 3.)

The perfection of our sense of *hearing* must in great measure depend on the practice of each individual. A knowledge of simple sensations cannot be transferred by description.

Chap. 2. *On the Physical Signs of the State and Action of the Thoracic Viscera.*—In the first section, we have a slight sketch of the variety of sound which will be yielded by striking gently with the fingers the different parts of the chest of a person in health. If the density of the organs contained in the thorax is changed by disease, the pectoral resonance will of course be modified.

"If, for example, a liquid or solid effusion take place in any part of the lungs or pleura, the corresponding portion of the chest will yield a dull, dead sound, and without that hollow resonance which is naturally produced by air underneath. On the other hand, when the aeriform contents of the cavity are increased beyond their usual proportion, as in pneumothorax and emphysema, the natural resonance may be increased to a degree that sounds quite tympanitic." (P. 18.)

As the practice of percussion requires some manual dexterity, and as on this, in great measure, depends the correctness of its indications, Dr. Williams offers a few observations on the best method of performing it.

"It is of very little consequence whether the patient be sitting or standing, or sitting up in bed, provided we hold in mind that all the sounds, bad and good, are rendered somewhat duller in the latter case by the vicinity of the pillows and bedclothes, which destroy the resonant echo accompanying sounds in more empty rooms. The same amount of difference may be perceived in eases before the appearance of the eruption, entirely from the peculiar odour of the patients.—Rev.

different rooms, when percussion is practised in the standing or sitting posture. In some cases of debility, and of painful disease, the patient can bear no other than the recumbent posture; and, in the parts where percussion can be practised, the sounds are somewhat more dull in these cases, from the deadening effect which the bed has on them. Thus warned, a little practice will enable the student to avoid error from these causes.

"The part on which percussion is practised should be covered with a linen or cotton garment, to render the stroke of percussion more equable, and to prevent its producing pain; and for this purpose a shirt or bed-gown kept on answers very well, if care be taken to keep it smooth and close on the surface by the fingers of the left hand." (P. 18.)

Every necessary detail upon this subject will be found in Laennec and other writers. In some cases in which the parietes of the chest are particularly tender, mediate percussion, in the manner recommended by M. Piorry, will be most proper. This is done by interposing a thin lamina of wood, horn, or ivory, on the part to be struck, so that, while the impulse of percussion is perfectly transmitted to the interior of the chest, it is so diffused on the surface covered by the lamina as not to produce pain. The indications obtained by percussion, although they only relate to the density of the parts, are of great value, and alone may sometimes, in the opinion of the author, detect diseases that all other signs leave in obscurity. But their importance and value are vastly enhanced, when they are combined with the more numerous and precise signs discovered by "*auscultation*," which forms the subject of the next section.

The signs of auscultation are those sounds produced in the chest, which may be heard by the direct or mediate application of the ear to its parietes. The author endeavours to trace these sounds to their physical causes, "and, by thus exploring the relations of diseases to certain and unchanging laws of natural philosophy, to place their characters beyond the doubtfulness and obscurity of sympathetic and sensatory signs." There is a considerable difference in the intensity of the sound of respiration in different individuals; and this depends partly on the thickness of the parietes of the chest, but principally on the degree of activity of the respiratory function. Dr. W. gives a general view of the physical signs of the state of the lungs; and, as all the phenomena noticed may be explained according to the laws of acoustics, it is presumed we shall not meet with any greater difficulty when examining them more minutely as the signs of particular diseases.

As, in many cases, there are objections to immediate auscultation, the stethoscope should be employed in general practice; and Dr. W. very truly observes, that well-regulated practice in the use of this instrument is worth a volume of directions and cautions. The mode in which it is employed is, however, briefly described.

Part II. *On the Physical Signs of Diseases in the Lungs and Pleura.*—The author has hitherto considered physical signs only with relation to the natural or physical state, and the general pathology of the lungs. He now enters upon the forms or characters that individual diseases present to the auscultator.

We are told that the pathological cause of *bronchitis,* or pulmonary catarrh, "is an inflammation and altered secretion of the mucous membrane of the bronchia;" that there are several varieties, and perhaps even species, of this disease, and that they pass insensibly into each other. Inflammation of the mucous membrane of the bronchi at first causes tumefaction, and partial obstruction of their calibre. The passage of air through the bronchial tubes is thus modified; vibrations are produced, and these tubes are converted into instruments of music. According to the extent and nature of the alteration in the structure of these parts we shall have various sounds, of which Dr. Williams endeavours to convey an idea, by comparing them to a *whistle,* a *horn* or *trumpet,* a *violincello,* or the *cooing of a dove.* The attempt to convey to those who have not had opportunities of hearing the various sounds produced by different derangements of the aerial tubes, by these forced and imaginative comparisons, is really as absurd as it is useless. When once heard, they will not easily be forgotten, but they defy a precise or useful description. The various kinds of *râle* have been so minutely described by Laennec, that we are not inclined to dwell upon the brief repetition with which Dr. Williams favors us upon this subject.

*Spasmodic Asthma.*—

"During the paroxysm the chest sounds ill on percussion, and the respiratory murmur is indistinct, even on the most forcible respiration. But if the patient, after holding his breath a little while, be desired to breathe again quietly, the spasm will be overcome as it were by surprise, and the entry of the air into the cells will be heard in a clear, and sometimes puerile, sound. This may be best effected in the manner recommended by Laennec, by desiring the patient to read aloud, or speak as many words as he conveniently can without taking breath, and then to breathe at his ease. But, after one or two inspirations, the spasm regains its hold, and the respiration becomes as dull as ever. The diminution of the respiratory noise here obviously proceeds from the obstruction opposed to the entry of air into the small bronchi and vesicles, by the tomic contraction of their muscular fibres. By the same contraction, the lungs are in a manner collapsed within the thoracic cavity, and the parietes of the chest, falling in with them, lose that sonorous elasticity produced by a fulness of aerial contents. The chest, thus contracted to the size of the collapsed lungs, may be compared to a drum, the parchment of which is pulled in by transverse strings. The free vibration is thus checked by these unyielding frena. Conceiving, as I do, that the contraction of the bronchial muscles is a sufficient cause of the phenomena of asthma, I gladly discard Laennec's hypothesis of the active dilatation of the bronchi, unsupported as it is by physiological fact, and opposed to all we know of animal dynamics.

"The dyspnœa produced by spasm of the bronchi is often of long continuance, and may to a certain extent become habitual. In such cases the system accommodates itself to the diminished supply of air, and the respiratory function is less called into action; but slight causes, either reproducing the want in the system, or increasing the spasm, will be sufficient to bring back the dyspnœa. Of the first class of causes are exertion, the sudden application of cold, &c.; of the second, depressing affections of the mind, and sympathetic irritations, produced by certain ingesta in the stomach and intestines. This second class includes usually those which originally produce the disease. I have seen a remarkable and exquisite case produced by the slow introduction of lead into the system; but such a form of saturnine neurosis is, I believe, rare." (P. 77.)

We pass over, without any apology, several of the succeeding sections, which, although they contain many proofs of the attentive observation of the author, are little more than brief surveys of doctrines and opinions more explicitly discussed by Laennec, Andral, and other writers.

*Phthisis Pulmonalis.*—Dr. Williams commences this chapter with an observation which does not appear to us perfectly correct. "The disease termed phthisis pulmonalis is produced by the formation of a particular matter called tubercle in the tissue of the lungs." Now, we apprehend that something more than the mere formation of tubercle is necessary to constitute the disease; for tubercle may exist for years without the appearance of any symptoms of pulmonary consumption. Neither do we apprehend that the existence of tubercle constitutes an essential part of pulmonary consumption, even taking the term in its strictest application. Catarrhal affection, long continued inflammation of the bronchi, are frequently marked by all the characteristic symptoms of phthisis, and will as fairly claim the addition of the term *pulmonary* as those cases in which tubercle exists in the tissue of the lungs. The definition of the author is therefore, in our opinion, inaccurate as to the fact it expresses, and too limited in its extent. Dr. Williams first traces the progress of the changes which morbid anatomy has shewn tubercles to undergo in the progress of the disease, and afterwards inquires into their nature and origin.

The error of framing systems or general plans of medical science upon any exclusive dogmas, is very properly and eloquently deprecated.

"It is no partial observer that can form for us a philosophical and comprehensive system of medicine. It is not the mechanist; for, although the body is a machine, it is much more. It is not the chemist; for, although the body is a laboratory, it is much more. It is not the vitalist; for the body is not disobedient to physical laws. It is not the humoralist; for the solids have also their specific properties. It is not the solidist; for the fluids may change of themselves, or be changed from without. It is not the empyric; for neither bodies, nor even the body, are always the same. Nor is it the morbid anatomist; for his dissections teach him little of causes, or of their relations with effects. It is to him who is all, and none of these; who views the animal body as a machine of its own kind, obeying physical and chemical laws in unexampled complication, and further disguised by a combination with others peculiar to living structure; and who, duly regarding all these powers, seeks, in a change in their relations, the causes and the cures of disease: it is to the PHYSIOLOGICAL PATHOLOGIST that I would look for the improvement of medicine; and to the combined exertions of many such, for the ultimate achievement of its greatest possible perfection." (P. 162.)

The volume concludes with a description of the "physical signs" of phthisis.

The greater part of this work is occupied by the repetition of doctrines and facts relating to percussion and auscultation, both mediate and immediate, which confessedly do not originate with the author. He has succeeded, however, in giving an air of profundity to his performance, by a philosophical investigation of the "physical signs" of pulmonary diseases. We cannot conceive that the execution of such a task can have been attended with much difficulty, and we candidly confess our fears that it will not materially conduce to our practical information. But a slight knowledge of the general laws of natural philosophy can be required to satisfy the student why percussion of the chest will produce different sounds according to the variation in the structure of the parts contained in it. An equally superficial knowledge of acoustics would make him acquainted with the rationale of the different phenomena discovered by the application of the stethoscope, although Dr. Williams had never deemed it necessary "to analyse its physical office."

The physical signs of diseases are certainly of quite sufficient importance to merit the careful attention of the practitioner. But we doubt the expediency or advantage of making them the subject of distinct dissertations. They should be studied in connexion with all the other characteristics of morbid action.

